# Cost-effective generation of precise label-free quantitative proteomes in high-throughput by microLC and data-independent acquisition

**DOI:** 10.1038/s41598-018-22610-4

**Published:** 2018-03-12

**Authors:** Jakob Vowinckel, Aleksej Zelezniak, Roland Bruderer, Michael Mülleder, Lukas Reiter, Markus Ralser

**Affiliations:** 1 Department of Biochemistry and Cambridge Systems Biology Centre, University of Cambridge, 80 Tennis Court Rd, Cambridge, CB2 1GA UK; 2 The Molecular Biology of Metabolism Laboratory, The Francis Crick Institute, 1 Midland Rd, London, NW1 1AT UK; 3 Biognosys AG, Wagistrasse 21, CH-8952 Schlieren, Switzerland; 4 Department of Biology and Biological Engineering, Chalmers University of Technology, Kemigården 10, SE-412 96 Göteborg, Sweden; 5 Science for Life Laboratory, Tomtebodavägen 23 A, 17165 Solna, Sweden

## Abstract

Quantitative proteomics is key for basic research, but needs improvements to satisfy an increasing demand for large sample series in diagnostics, academia and industry. A switch from nanoflowrate to microflowrate chromatography can improve throughput and reduce costs. However, concerns about undersampling and coverage have so far hampered its broad application. We used a QTOF mass spectrometer of the penultimate generation (TripleTOF5600), converted a nanoLC system into a microflow platform, and adapted a SWATH regime for large sample series by implementing retention time- and batch correction strategies. From 3 µg to 5 µg of unfractionated tryptic digests that are obtained from proteomics-typical amounts of starting material, microLC-SWATH-MS quantifies up to 4000 human or 1750 yeast proteins in an hour or less. In the acquisition of 750 yeast proteomes, retention times varied between 2% and 5%, and quantified the typical peptide with 5–8% signal variation in replicates, and below 20% in samples acquired over a five-months period. Providing precise quantities without being dependent on the latest hardware, our study demonstrates that the combination of microflow chromatography and data-independent acquisition strategies has the potential to overcome current bottlenecks in academia and industry, enabling the cost-effective generation of precise quantitative proteomes in large scale.

## Introduction

In basic biological and biomedical research, mass spectrometry-based proteomics has emerged as a prime technology for identifying and quantifying proteins, determining activity, turnover, modification state, and closing the gaps in structural biochemistry^1–3^. Proteomic technology has progressed to the extent that, for low sample numbers, a broad coverage of the proteome is achieved^4,5^.

However, the same proteomic technology is far less frequently applied for the analysis of large quantitative sample series. The throughput and robustness required to analyse hundreds of proteomes in one experiment has been achieved by a few laboratories worldwide, is however by no means standard. The acquistion of large numbers of proteomes is currently associated with high costs, driven to a considerable extent by instrument time. In this sense, proteomics lags behind the other ‘omic’ disciplines, i.e. genomics, transcriptomics and metabolomics, where the precise analysis of large and very large sample series has become more regularly achieved. Large and intrinsically comparable sample series are however increasingly demanded also from proteomic experiments. Such sample series are required for data driven biology, clinical research and for diagnostic applications^6^.

Techncially, some key objectives substantially differ between proteomic experiments that deal with small compared to large sample series, and make it difficult to scale up any proteomic experiment. In small-scale experiments the number of peptides quantified, or proteomic depth, is typically a key parameter to benchmark the success of a proteomic experiment. In large sample series however, the effects of measurement noise amplify, batch effects become prevalent, and stochastic elements reduce the number of consistently quantifiable peptides. In large scale applications, the precision as to how well the identified peptides are quantifiable, is hence a key parameter. Difficulties in maintaining quantification precision when scaling up a quantitative proteomic experiment have several sources. One of them is the typical proteomic nanolitre flow rate chromatography (nanoLC). While handling of the typical proteomic nanoflow chromatography has become simpler, it still requires expert knowledge to be maintained robustly. The low flow rate and small capillary diameters as required in nanoLC systems are susceptible to all sorts of technical distortions that regularly create major batch effects. Typical reversed-phase columns used in nanoLC need to be exchanged after a maximum of a few hundred injections, while the low flow rates render it a challenge to maintain an electrospray fully stable over long measurement periods. As a consequence, batch effects detected in large sample series are often stronger than the protein expression differences between the typical samples^2,7^.

Other constraints emerge from the situation that proteomes are complex by nature. This introduces stochastic elements through ‘undersampling’, when the acquisition speed of a mass spectrometer is slower than the number of co-eluting analytes. Undersampling reduces the number of consistently quantifiable peptides the more samples are measured. There are several developments to overcome undersampling. These include to computationally impute missing values or to record proteomes in a data-independent manner^2,8,9^. However, in the end it is physical limits that make the detection of every single peptide in every single sample a challenging task.

A decade of proteome research has revealed important molecular details about function and regulation of the proteome. This biological information can be exploited to tailor a new generation of proteomics workflows to obtain the maximum biological information despite technical limits. Most importantly, many proteomic experiments have shown typical proteomic responses affecting dozens to hundreds of proteins in parallel, so that not all of them have to be quantified in order to detect a precise biological signature. This allows a substantial amount of biological information to be retrieved from just a subsets of proteins, sentinels or antibody-detectable protein markers, for instance^10–12^. However, a difficulty remains that the quantitative concentration changes detected on the proteome level, in relative terms, are often smaller than the ones detected at the transcriptome or metabolome level and, therefore, require more precise measurements^13^. For many new applications of proteomics, like the ones which concentrate on pattern detection and data-driven systems biology, the ultimate goal of achieving full proteome coverage has become less urgent compared to the rapidly increasing need for achieving high quantitative precision that allows small concentration changes to be reliably detected across hundreds to thousands of samples. This demands workflows that are cost-effective, robust and easy to handle to the extent that they can be established by a large number of laboratories world-wide^10,14–16^.

Taking the advancements of proteomic technology and the main needs for data-driven biology into account, we combine several strategies to conceive a proteomic platform complementing other proteomic methods specifically for the label-free protein quantification in large sample series. The platform combines microflow chromatography, an analytical flow rate ion spray source (TurboV source, Sciex), data independent (SWATH) acquisition on a penultimate generation QqTOF instrument (TripleTOF5600, Sciex). We put particular emphasis and established computational correction strategies for retention time shifts and non-linear batch effects that emerge in the same sample size. Furthermore, being able to determine the correlation of peptides across many samples, we exploit this information to improve the selection of quantifier peptides, typically conducted over abundance only. Even without being reliant on the newest or most expensive mass spectrometry nor LC hardware, we achieve the precise and consistent quantification of thousands of peptides across hundreds of proteomes. We report precision values consistently determined at large scale that are typically seen in highly controlled, small-scale proteomics experiments.

## Results

Many of the key proteomic developments over the last decade have been concentrated on ion trap instruments due to their high sensitivity and resolution. However, due to their acquisition speed and high dynamic range, data independent acquisition workflows have been developed frequently on Quadrupole time of flight (qTOF) instruments, and include the successful DIA workflows MS^E^^17^ and SWATH-MS^18^. Indeed, fast and increasingly sensitive qTOF instruments are available from multiple manufacturers and are popular in high-throughput laboratories due to their fast acquisition speed.

To be comparable with previous SWATH-MS developments, and to enable laboratories that have previously invested in DIA technology to implement our workflow, we use a conventionally sensitive, yet fast (100 HZ acquisition) QqTOF mass spectrometer (TripleTOF 5600^19^, SCIEX) on which the SWATH-MS acquisition method was originally developed^18^. The TripleTOF 5600 is not the newest and most sensitive mass spectrometer. Therefore, our study also serves as a benchmark as to what is realistically achievable without access to the newest and most expensive hardware. Not being dependent on the latest hardware can reduce costs and implementation burdens dramatically, and is important to enable broad access to large-scale, quantitative workflows. Along this line, in order to avoid specialist hardware dependency on the LC side, we combined this mass spectrometer with typical proteomic nanoLCs (NanoAquity, Waters, UK and nanoLC425, Eksigent, USA) that have been converted into microLCs through the exchange of capillaries (Waters) or flow modules (Eksigent). A further reduction of cost, and in parallel increased robustness, is also achieved through the use of microflow columns and analytical flow rate ionspray source. In our experience microflow columns block less frequently, and ideally last for years. On samples as prepared and analysed as in this study, a nanoLC column lasts typically up to 200 injections, while on a comparable set-up and samples, microLC columns last for about 3000 injections. (Please note that like in any chromatographic method, these values vary dependent on sample quality, LC method and hardware used). By reducing batch effects caused by exchange and/or blockage of columns, this situation reduces hands-on time (columns need be exchanged less frequently and, therefore, the electrospray needs fewer readjustments), and reduces the costs incurred through column purchases by up to 90–95%.

**Figure 1:**
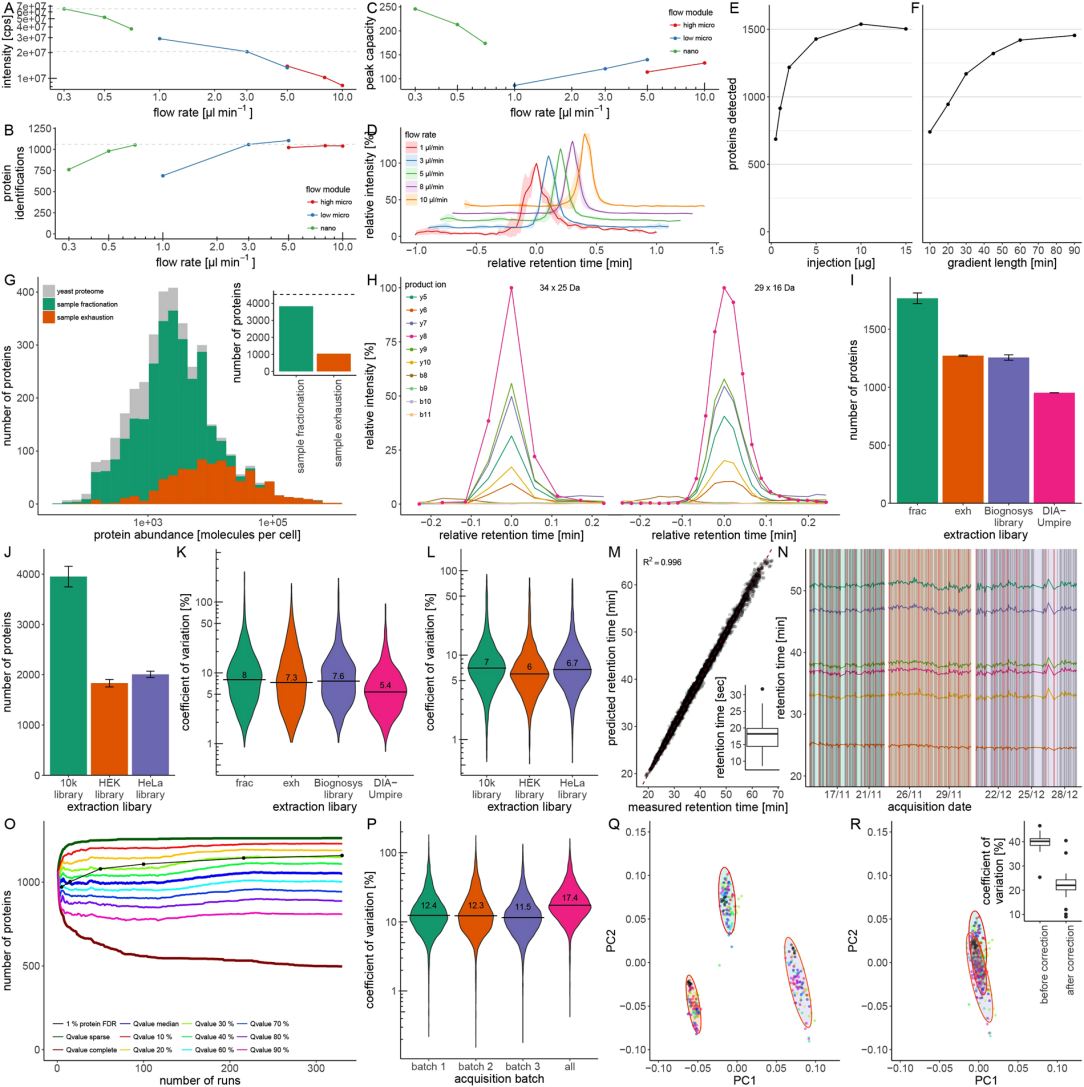
(**A**) Dependency of signal intensity on flow rate in a proteomic experiment. Combined intensities of standardized peptides (iRT) determined using nano, low-micro, and high-micro flow regimes on an Eksigent 425 LC system equipped with three respective flow modules and recorded on a TripleTOF5600 mass spectrometer. Signal intensity is a function of the dilution rate, with a factor of 0.3 between 0.3 µL/min and 3 µL/min. (**B**) Dependency of protein identifications on flow rate in a proteomic experiment. The number of detectable proteins by DDA were determined using nano, low-micro, and high-micro flow regimes on an Eksigent 425 LC system equipped with three respective flow modules and recorded on a TripleTOF5600 mass spectrometer. Number of identified proteins increased moderately with flow rate. (**C**) Peak capacities on a proteomic microLC set-up. Peak capacity was calculated from the measurement of standard peptides on the same chromatographic setup, varying the flow rate from 300 nL/min to 10 µL/min. Peak capacities of microLC increase with flow rate. (**D**) Peak characteristics on a proteomic microLC set-up. Average precursor peak shapes of 5 iRT peptides determined using flow rates of 1–10 µL/min on an Eksigent 3C18-CL-120 column. Chromatography is stable and reproducible in flow rates >1 µL/min. Shaded areas represent standard deviation of signal intensity. (**E**) 2 µg tryptic protein digest is sufficient to quantify >1200 yeast proteins in a single injection in microLC-SWATH-MS. Tryptic digests obtained from 1–15 µg of yeast whole proteome extracts^33^ were injected and separated using a 60 min water to acetonitrile gradient at a flow-rate of 3 µL/min. 914 proteins were quantified with 1 µg, 1219 proteins with 2 µg, 1428 proteins with 5 µg and 1504 proteins with 15 µg digested protein. (**F)** A 30 min LC gradient is sufficient to quantify >1000 yeast proteins in a single injection in microLC-SWATH-MS. A tryptic digest derived from 5 µg yeast protein was injected and separated using water-to-acetonitrile chromatographic gradients of 10–90 min at a flow-rate of 3 µL/min. Extraction of the SWATH spectra yielded quantifiable peptides for 740 proteins (10 min), 946 proteins (20 min), 1170 proteins (30 min), 1322 proteins (45 min), 1420 proteins (60 min) and 1455 proteins (90 min). SWATH-MS data was extracted in Spectronaut 8.0 using a spectral library generated by yeast proteome prefractionation. (**G)** The coverage of the yeast proteome by microLC-based proteomics upon prefractionation. A yeast tryptic digest obtained from BY4741-pHLUM^32^ was first separated by high pH reverse phase chromatography on an analytical HPLC and then analyzed in DDA mode with *m*/*z* (gas phase) fractionation at 3 µL/min flow rate. In the sample exhaustion approach, the same digest was instead injected repeatedly until protein identification was saturated. When comparing the proteins identified in both approaches with the abundances of yeast proteins as measured by fluorescence microscopy^21^, the most abundant proteins were consistently identified, while proteins with low expression levels were only identified upon pre-fractionation. Inset: In total, 3822 (84%) or 1037 (23%) out of 4517 expressed yeast proteins^21^ were identified using either microLC-SWATH method, respectively. (**H**) Peak representation in microLC-SWATH-MS. Extracted ion chromatogram (XIC) of the peptide TPVITGAPYYER recorded in microLC-SWATH mode using either 34 × 25 m/z or 29 × 16 m/z windows, respectively. A conventional (nanoLC-optimized) SWATH setting^25^ of 34 × 25 m/z with a cycling time of 3.3 s leads to a coverage of 5 points per peak. When limiting the mass range covered to 400–850 m/z which contains precursors for 96% of proteins, and reducing accumulation time to 40 ms, cycling time is 1.3 s to cover microLC chromatographic peaks by >11 data points, (**I)** Different strategies to construct SWATH spectral libraries and their application in microLC-SWATH-MS. A yeast tryptic digest was analyzed using microLC (0.3 mm × 250 mm Triart-C18, 3 µL/min, 60 min gradient) SWATH-MS by repeated (9×) injection of a tryptic digest derived from 10 µg yeast protein. Data was processed with Spectronaut 8.0 using SWATH libraries generated by either sample fractionation (*frac*), sample exhaustion (*exh*; matrix-matched library), using a spectral library recorded in an unrelated lab and instrument set-up (Biognosys library), or with a library generated by DIA-Umpire without physically recording a separate spectral library. Data analysis on the basis of the fractionation allowed quantification of 1766 proteins, the exhaustion library quantified 1271 proteins, the unrelated SWATH library 1256 proteins, and DIA-Umpire 952 proteins. DIA-Umpire yielded the lowest variability. (**J)** Human protein quantification using microLC-SWATH. A tryptic digest of a whole-cell protein extract from human K562 cells was analyzed using microLC (0.3 mm × 250 mm Triart-C18, 3 µL/min, 60 min gradient) and coupled to a TripleTOF5600 MS operating in SWATH mode by analysing 3 µg tryptic digest six times. Data was processed with Spectronaut 8.0 using a SWATH library obtained from the SWATHAtlas repository^31^ (10k library), or using SWATH libraries generated by repeated analysis of HEK293 or HeLa cell extracts (Spectronaut repository). Data extraction by using the rich library quantified 4169 K562 proteins, while 2031 proteins when using a library generated from HEK293 cells, and 1906 using a HeLa library, respectively. (**K)** Precision of yeast protein quantification using microLC-SWATH-MS. Signal variability (expressed as fold change) of 677 proteins present in all datasets was compared throughout nine replicates. Median coefficients of variation are between 7.3% and 8% for libraries generated using respectively fractionation (*frac*) and exhaustion (*exh*) approach, 7.6% for an unrelated yeast library, and 5.4% for DIA-Umpire. (**L)** Technical variability of human protein quantification is low in microLC-SWATH-MS. Signal variability (expressed as fold-change) of 726 proteins present in all datasets was compared throughout the six replicates. (**M**) Retention time stability microLC-SWATH-MS over 327 yeast whole-proteome acquisitions in three defined batches. Correlation between measured apex retention time and predicted retention time. Shown is a representative yeast sample acquired in SWATH mode. Inset: Mean retention time standard deviation of 6 iRT peptides across 327 injections is 17.7 s. (**N)** Retention time stability in large sample series as measured by microLC-SWATH-MS. 327 yeast tryptic digest samples spiked with iRT peptides were analyzed by microLC-SWATH-MS in three batches in a net acquisition time of 16 days (grey vertical lines). Retention times of iRT peptides are shown over time (colored lines), and retention time coefficient of variation for all peptides is lower than 2% over the whole period. Red vertical lines indicate the interspersed quality control samples (QC) sample measurements needed for batch correction. (**O**) Completeness of a large SWATH dataset is well represented with median Qvalue filtering. The number of proteins in a SWATH dataset of 1 to 327 yeast samples was determined with either sparse or complete Qvalue filtering, Qvalue percentile or Qvalue median filtering as implemented in Spectronaut software (coloured lines). The number of proteins was also determined when applying a 1% protein FDR filter (black line). (**P)** Quantification precision in large sample series as measured by microLC-SWATH-MS. Coefficient of variation for fold changes of 8686 peptides was calculated in batch 1 (green), batch 2 (orange), batch 3 (purple) or across batches (magenta) prior to batch correction. Intra-batch CVs were around 12%, while variability over the entire 27 day period was 17.4%, as calculated from the repeated measurement of QC samples (as in (N)). (**Q)** The quantification of 327 yeast proteomes before batch correction. 38 yeast strains were grown in three batches, and each batch was acquired as three technical replicates in SWATH-MS together with 10–12 evenly distributed QC samples. In a PCA, proteomes cluster according to the acquisition batch, with color-coded technical replicates clustering together. (**R**) The quantification of 327 yeast proteomes after batch correction. After batch correction based on the combined quality control sample profiles, clustering according to batches is reduced, and proteomes cluster according to the color-coded yeast strain. Inset: Median coefficients of variation of peptide intensities between all 9 replicates of each strain are 39.7 ± 3.2 before batch correction and 22.3 ± 5.4 after batch correction.

After having set-up the platform, we started with an evaluation of the relationship between sensitivity and flow rates on the combined nanoLC and microLC chromatographic set-up using the Eksigent LC by first operating the mass spectrometer, like in a conventional proteomic experiments, with a data-dependent acquisition (DDA) workflow. By varying flow rates from 300 nL/min to 10 µL/min, on 75 µm (0.3–0.7 µL/min) or 300 µm (1–10 µL/min) inner diameter columns, we compared the signal intensities of spiked standardized peptides (iRT standards^20^) and the number of quantified proteins out of an unfractionated yeast whole proteome tryptic digest. While the 30-fold stepwise increase in flow rate reduced the total signal intensities by a factor of seven (Fig. 1A), it did not affect the number of detected proteins in yeast lysates with FDR cutoff <0.01 (Fig. 1B). Furthermore, while peak capacity decreased with increasing flow rates on the nanoflow setting, in microflow mode, chromatographic peak capacity increased with flow rate (Fig. 1C). A good compromise, considering total signal intensity, peak capacity and chromatographic quality was found at flow rates between 3 µL/min and 5 µL/min. While chromatographic quality was stable at a broad range of flow rates (Fig. 1D), at 3 µL/min signal intensities were reduced only by a factor of 3.5 compared to nanoLC-MS/MS operating at 300 nL/min (Fig. 1A, dotted lines). Next, this moderate decline in sensitivity could be compensated by exploiting the higher sample capacity of the microLC columns, which allow loading of up to 15 µg whole-proteome tryptic digest (up to 10× the amount that can be separated in nanoflow chromatography). In DDA mode, the analysis of an unfractionated yeast tryptic whole proteome digest led to the detection of >1200 proteins (1/4th of the yeast proteome) with as little as 2 µg unfractionated tryptic whole-proteome digest per single injection (Fig. 1E). Hence, microflow chromatography can be applied to quantitative proteomics without necessarily causing a substantial decline in protein identification numbers. A summary of all performed experiments, acquisition modes and settings is presented in Supplementary Table 1.

At this point it should be noted that, while more peptides are injected for microflow-chromatography, not more starting material is required for generating the samples. Due to handling difficulties (i.e. difficulties to filter and pH-adjust small volumes of liquid), typical proteomic sample preparation methods yield ~5–20 µL of tryptic digest. On nanoLC-MS/MS settings, 1 µL to 2 µL of the digest is typically injected, and 5–10 µL of the same digest is injected in the microLC setting. Thus, our microLC-based workflow does not require more input sample material. Instead a lower amount of injection replicates can be run per sample. For most applications of quantitative proteomics however, multiple injection replicates are not required, as statistical methods require biological or at least full technical replicates. Injection replicates and are hence typically recorded only for method development purposes or for the generation of spectral libraries, but not on large sample series. Indeed, in practice, microLC-based proteomics may be able to handle more diluted samples as derived from highly limiting starting material, for the simple reason a higher spectrum of injection volumes can be handled chromatographically.

We continued with an optimisation of the microflow chromatography, focussing on finding a good compromise between number of identified analytes and gradient length for high throughput applications. While protein identification capacity increased with gradient length as expected, 30 minute microflow gradients were sufficient to identify >1000 yeast proteins (FDR <0.01) in a single injection of an unfractionated whole-proteome tryptic digest as analyzed by DDA (Fig. 1F). In combination with high pH reversed phase chromatography prefractionation, the setup identified 3822 proteins, or 85% of all expressed yeast open reading frames as detected by GFP fusion and fluorescence microscopy^21^. Microflow-SWATH-MS is therefore competitive in regards to other proteomic technologies that use nanoflow chromatography on instruments of a comparable generation^22^.

The 15% of non-detected proteins are, for the most part, proteins of low concentration, reflecting a proteomics-typical abundance bias (Fig. 1G). On a newer generation of ion trap mass spectrometers, yeast proteomic depth did, upon extensive fractionation, go beyond these detection limits^23^. These results imply that the obtained protein number values are not yet exhausted and will increase when the workflow is optimized for increasing proteomic depth instead of sample numbers and robustness, or when using a more sensitive mass spectrometer (not the focus of this study).

Focussing on the chromatography, we obtained chromatographic peaks with an average full-width at half maximum (FWHM) of 12 seconds. While narrow peaks are generally desirable in chromatography, in proteomics they amplify the problem of undersampling, a situation that emerges when more peptides elute at any unit in time than the mass spectrometer can process^24^. Data-independent acquisition (DIA) strategies, like SWATH-MS where all precursors falling into an isolation window are fragmented simultaneously and chromatograms are reconstructed computationally post-acquisition, have been designed to overcome this problem^25^. However, cycle times of conventional SWATH-MS were optimised for typical nanoLC-applications and are in the range of 3 seconds. They would cover a 12 sec wide peak with only 4–6 data points, too little for accurate peak representation in precise quantification experiments (Fig. 1H). The SWATH regime was accelerated for 12 sec FWHM peaks by reducing the cycle time to 1.3 s. For this, the isolation window dwell time was reduced to to 40 ms, which did not cause a notable loss in signal intensity or number of peptide identifications (Suppl. Fig. 1). To further accelerate the acquisition cycle, we compromised and limited the segmented acquisition to record only the precursor-rich mass range between 400–850 m/z. This mass range covers 85% of precursors and enables the quantification of 96% of proteins (Suppl. Fig. 2). Upon these modifications, our SWATH-MS method covered the typical microLC chromatographic peak with a critical number of 8–12 data points, yielding accurate peak representation for the application of fast chromatography (Fig. 1H).

Protein identification numbers and the quality of quantification are both dependent on the recorded data but also, specifically in DIA acquisition, on the spectral libraries used to extract the data. Therefore, we processed the datasets using different library generation strategies. SWATH libraries were generated in Spectronaut^26^ using proteome prefractionation (following standard approaches^25,27^), repeated injection of a sample mixture matching the actual sample matrix (‘exhaustion’), or by pseudo-MS/MS correlative precursor-fragment feature extraction using DIA-Umpire^28^. The spectral libraries were generated following a previously optimized procedure^27^ at <1% FDR using a combination of X! Tandem^29^ and Comet^30^ search engines. The libraries were used to quantify peptides in an unfractionated, whole-cell *Saccharomyces cerevisiae* tryptic digest, of which 10 µg were separated on 60 min microLC gradients at a flow rate of 3 µL/min. By using the spectral library created by prefractionation, we quantified 1766 ± 46 yeast proteins using 34 × 25 m/z SWATH windows, or 1422 ± 53 proteins when using 29 × 16 m/z windows (Fig. 1H and Suppl. Fig. 3). The library generated by repeated injection of the same digest (exhaustion) yielded the quantification of 1271 ± 5 and 1157 ± 13 proteins, a similar performance compared to data-extraction with a totally independently created and publicly available SWATH library generated by nanoLC-MS/MS^26^. Although generated using another chromatography regime, this library quantified 1256 ± 23 and 1118 ± 26 proteins on the microflow datasets, respectively. Without the need for a separately acquired spectral library, on this sample DIA-Umpire quantified 952 ± 0 and 890 ± 2 proteins (Fig. 1I and Suppl. Fig. 3). Peptide quantification numbers followed similar trends (Suppl. Figs 4 and 5). In parallel, we tested the performance of microLC-SWATH-MS on a standardized whole-proteome human cell line (K562) tryptic digest, by extracting data using three publicly available spectral libraries generated by combining multiple tissues and fractionation^31^ or by repetitive injection of tissue-specific cell digests of HEK293 or HeLa cells (Spectronaut^26^ repository). MicroLC-SWATH-MS achieved quantification of 3951 ± 205, 1832 ± 74 and 2007 ± 63 proteins, respectively, out of single-injections of the unfractionated K562 protein digest, with peptide numbers following the same trend (Fig. 1J, Suppl. Fig. 7).

The implementation of microLC-SWATH-MS yielded precise quantities for label free proteomics, both in small scale and large scale experiments. In small scale, the median coefficients of variation (CVs) for replicate injections of the yeast samples in all acquisition strategies and analysis libraries were 5.4–8.8% (Fig. 1K and Suppl. Fig. 6) and 5.5–7% for the human cell line (Fig. L) and Suppl. Fig. 8). The precision was largely similar over the full dynamic range spanning five orders of magnitude (Suppl. Figs 9 and 10). Interestingly, proteins identified by DIA-Umpire, which in our samples were lower in number compared to other approaches, yielded a higher precision in the quantification experiments (Fig. 1K). This could be related to a better signal-to-noise ratio of high abundant analytes, or to the highly abundant part of the proteome being generally more stable. Indeed, we also detect the abundance bias in the peptides identified by DIA-Umpire, indicating its the quantification of more abundant peptides that results in more precise values (Suppl. Fig. 11).

In order to determine the performance characteristics of microLC-SWATH-MS for the intended application of acquiring large numbers of proteomes for data driven biology, we conducted two large studies to optimize strategies for retention time and batch correction, as well as peptide selection. In the first, we analyzed 296 proteomes of *Saccharomyces cerevisiae* strains in the BY4741-pHLUM background^32^. 38 yeast strains, each with a single gene deletion, were grown in nine replicates to exponential phase, sampled, and processed by a protocol using the Rapigest detergent (Waters, UK) as reported earlier^33^. Including quality control (QC) samples, this benchmark span over 327 whole-proteome samples, recorded in three batches upon coupling the QTOF mass spectrometer to a commercial nanoLC system (nanoAcquity, Waters) that had been converted to a microLC by exchanging capillaries of larger diameter. Even before applying retention time normalization, the converted nanoLC yielded highly stable retention times in microflow (standard deviation of apex retention times 17.7 s in 60 min gradients over the 327 runs, Fig. 1M, inset). This value was further improved using linear retention time normalisation using standard peptides^20^, upon which an excellent agreement with theoretically expected retention times was achieved (Fig. 1N). Furthermore, to address the batch effects that unavoidably confound quantitative proteomics when conducted in large scale^34,35^, we established a batch correction strategy that repetitively includes a mixture of a combined sample (QC control) every 10–12 injections. Then, we applied an empirical Bayes framework^36,37^ to correct data for observed batch effects associated with acquisition dates by specifying covariates. Batches spanning 100 to 116 whole-proteome acquisitions were each acquired over a period of around 9 days (net acquisition time 6 days). Analysing the proteomes using the spectral library created by sample exhaustion and containing 1323 proteins, yielded the typical quantification of 1212 ± 100 proteins in the average sample.

A typical problem of large-scale data analysis is that, ideally, it should yield the consistent quantification of a given analyte across all samples. Removing all precursors that have one or more values with >1% FDR in any of the 327 proteomes (*complete* filtering) reduces the number of proteins to 497. However, this procedure artificially reduces proteome depth, as it over-rates the FDR in one sample over the recurrence of a signal in hundreds of samples. Filtering with a Qvalue percentile of 10% is sufficient to increase the number of consistently quantified peptides by almost 50% (Fig. 1O). On the other hand, *sparse* filtering, i.e. retaining every precursor that has at least one value with >1% FDR, artificially inflates the dataset. In our hands, the best compromise was found when filtering by the median Qvalue, which gives both consistent results and retains a high number of quantified proteins (Fig. 1O). Indeed, when also evaluating proteome depth by controlling protein FDR, we found that median Qvalue filtering is more conservative than controlling protein FDR^38–40^.

Finally, we evaluated the quantitative precision of our workflow, which is arguably an equal or even more important benchmark compared to peptide identification numbers for judging the quality of large scale proteomics experiments produced for data-driven applications. For this, we determined coefficient of variation (CV) values for the obtained peptide quantities. The median CVs of the peptides quantified in QC sample (repeated injections over the whole experiment) were 12% within a batch, while 17.4% between batches (Fig. 1P). This characteristic equals, or even exceeds, precision values typically obtained in label and label-free proteomic experiments, even when conducted in small scale^25,41–43^.

In order to also demonstrate the retention of the biological variability upon batch correction, we analysed the differences between the technical CVs and total CVs, which include both technical and biological variability. Our batch correction method reduced the total signal variability by 43%, which largely corresponds to the batch effect, while maintaining the specific individual strain’s proteome profile within the batch, as illustrated by the confidence boundary in a principal component analysis (Fig. 1Q,R, Suppl. Figs 12 and 13). Eventually, the average variation of peptides quantifying >1100 proteins across the 327 samples, is determined with a median CV of 22.3 ± 5.4% (Fig. 1R, inset).

In parallel we tested the platform to see whether we can distinguish differences in proteomes of so called redundant biological signalling molecules by exploiting semi-targeted feature extractions from data-independent mass spectrometry data. For this benchmark, we selected all yeast strains deleted for a protein kinase that are viable in minimal medium^32^. We measured their proteome by microLC-SWATH-MS in exponential growth phase, and extracted the quantities of 289 metabolic enzymes, as these constitute one of the largest functional class of proteins in the high-abundant fraction of the proteome. In order to yield precision on the enzymome dataset, we selected the peptides for quantification not by abundance, but by highest pairwise correlation. This strategy assumes that all peptides that are derived from the same protein are suitable for quantification; this strategy excludes peptides that behave inconsistently across the measured samples, i.e. as they are differentially posttranslationally modified. We used a correlation-dependent peptide selection strategy, as developed by us earlier in combination with an MS^E^ workflow^13^. An analogous strategy is used in the mapDIA workflow, for example^44^. Then we optimized the batch correction strategy for the semi-targeted approach. We used parametric adjustment of the raw signals as in supervised surrogate variable (SVA) analysis^37^. SVA was applied without specifying experimental factors^45^, using 50% of least variable peptides as controls. Estimated surrogate effects were regressed out from the peptide signal. Finally, for each protein, the signals of all peptide groups were geometrically averaged. Following this semi-targeted strategy, 289 metabolic enzyme levels were quantified across 397 measured proteomes over a period of four months. The median quantitative precision (CV values) accessed from the QC samples that we injected over the duration of the experiment, was calculated to be 19%. Hence, microflow-SWATH-MS allows the precise semi-targeted quantification of large selected protein groups over hundreds of samples. A manuscript describing the biological meaning of enzyme abundance changes as measured in all viable yeast kinase knockout strains is submitted for publication elsewhere (Zelezniak *et al*., Submitted, *Cell Systems*, December 2017).

In conclusion, we show that microLC-based proteomics is able to achieve broad protein identification characteristics. In combination with a microflow-optimized data independent acquisition workflow, a platform is obtained that can capture a quantitative snapshot of a yeast or human proteome in an hour or less, and at the same time maintain high quantitative precision that remains consistent over months and over hundreds of proteomes. The performance characteristics are illustrated by the untargeted and semi-targeted quantification of proteins in >750 yeast proteomes acquired. We yielded a quantitative performance, expressed as lower than 20% average CV across hundreds of samples recorded over a five month acquisition period. It is important to mention that due to a more exhaustive usage of the created tryptic digests, the workflow does not need more starting material compared to conventional (nanoLC) based proteomic workflows, and is economic by not being dependent on the latest instrument hardware and the use of microflow-columns that have longer run times compared to their nanoLC-counterpart, reducing both material costs as well as hands-on time, while increasing throughput. Broad access to large and precisely measured sample series is the foundation for data driven systems biology that enables application of machine learning approaches to uncover so far missed biological patterns^46^. It further empowers the analysis of large time-series and cohorts of comparative studies in basic and clinical research, e.g. to enable predictive diagnostics. Indeed, the high flow rate renders microLC-SWATH-MS a highly robust proteomic technology with low instrument downtime and maintenance cycles, increasing data quality simply through minimizing instrumental bias and leading to higher productivity and cost effectiveness.

## Materials and Methods

### Materials, solutions and reagents

Chemicals and reagents were obtained from Sigma, UPLC/MS grade chromatographic solvents from Greyhound, unless stated otherwise.

### Sample preparation for mass spectrometry

A standardized yeast sample was generated by growing the prototrophic *Saccharomyces cerevisiae* strains YSBN1^47^ or BY4741-pHLUM^32^ in Yeast Nitrogen Base (YNB) medium without amino acids containing 2% glucose until mid-exponential phase. Cells were harvested by centrifugation and snap-frozen in aliquots equaling 10 OD_600_ units. Sample preparation was performed as described^33^ with the following modifications. Cells were broken by bead shaking with 200 µL 0.05 M ammonium bicarbonate in a FastPrep instrument (3 × 30 s, 6.5 m/s, 4 °C), and cell pellet after centrifugation was re-extracted with 200 µL lysis buffer (0.1 M NaOH, 0.05 M EDTA, 2% SDS, 2% 2-mercaptoethanol) for 10 min at 90 °C, and again for 10 min at 90 °C after addition of 0.1 M acetic acid. Combined supernatants were precipitated using 10% TCA, and processed further according to the RapiGest protocol^33^. Protein concentration before digest was determined using Pierce BCA assay kit (Thermo), and adjusted to 2 µg/µL with 0.2% RapiGest SF (Waters) in ABC. MS compatible human protein digest from K562 cells was obtained from Promega.

For library generation using pre-fractionation, 1 mg of yeast tryptic digest was separated by high pH reverse phase chromatography on a Waters ACQUITY instrument. A reverse phase column (Waters, BEH C18, 2.1 × 150 mm, 1.7 µm) was utilized in combination with a 20 mM ammonium formate to 20 mM ammonium formate/80% ACN gradient, collecting 33 fractions. Before analysis, samples were spiked with 0.5 × HRM kit (Biognosys). QC samples were prepared as a mixture of 10% of each individual sample.

### Chromatography

Chromatographic separation was performed either on an Ekspert NanoLC 425 system (Eksigent/SCIEX) for combined nano and micro flow analysis, or a nanoACQUITY system (Waters) for microflow-only sample series. In nano flow, the NanoLC 425 system was equipped with a nanolitre flow module, and samples were first loaded onto a trap column (Chrom XP C18–3µm, 0.12 nm, 0.35 × 0.5 mm) by isocratically running the system at a flow rate of 5 µL/min for 6 min with 0.1% formic acid (FA) in water. Peptides were then eluted onto the analytical column (3C18-CL-120, 3 µm, 0.12 nm, 0.075 × 150 mm, Eksigent) and separated on a linear gradient of 2–30% 0.1% FA in acetonitrile (ACN) in 25 min. For microlitre flow rate chromatography, the same system was equipped with a low microlitre flow module (1–5 µL/min) or high microlitre flow module (5–10 µL/min) and set up for direct injection onto an analytical column (3C18-CL-120, 3 µm, 120 Å, 0.3 × 150 mm, Eksigent). Separation was performed on a linear gradient of 2–30% 0.1% FA in ACN in 25 min. For microlitre flow rate chromatography on the nanoACQUITY system, the sample manager was set up in direct injection mode and equipped with a Triart C18 column (0.12 nm, 3 µm, 0.3 mm × 250 mm, YMC). After injecting samples onto the analytical column, peptides were separated on linear gradients detailed in Suppl. Table 2.

### Mass Spectrometry

SWATH data was recorded on a Tandem Quadrupole Time-of-Flight mass spectrometer (TripleTOF5600^19^, SCIEX) coupled to either a Nanospray III Ion Source (SCIEX) or a DuoSpray Analytical Ion Source (SCIEX), controlled by Analyst software (v.1.6). For nanoflow, the ion source was equipped with 10 µm SilicaTip electrospray emitters (New Objective) and parameters were as described^33^. For microflow, the ion source was equipped with a 25 µm TurboIonSpray probe (SCIEX), and parameters were as follows: ISVF = 5500, GS1 = 10, GS2 = 0, CUR = 25, TEM = 100.

To acquire spectral libraries, the mass spectrometer was operated in information-dependent acquisition (IDA) and high sensitivity mode, with first a 250 ms TOF MS survey scan over a mass range of 400–1250 m/z, followed by 100 ms MS/MS scans of 20 ion candidates per cycle with dynamic background subtraction. The selection criteria for the parent ions included the intensity, where ions had to be greater than 150 cps, with a charge state between 2 and 4. The dynamic exclusion duration was set for 1 s. Collision-induced dissociation was triggered by rolling collision energy. For generation of SWATH libraries by sample fractionation, precursor-rich fractions were further injected twice, with online gas phase fractionation between the mass ranges of 400–650 m/z and 650–1250 m/z. For data-independent acquisition, the instrument was operated in SWATH mode with selection windows detailed in Suppl. Table 3 for mass ranges of 400–1250 or 400–850 m/z. With accumulation times of 100 and 40 ms cycle times were 3.3 s and 1.3 s, respectively.

### Data analysis and SWATH library generation

All SWATH assay libraries generated in this study were built following the protocol by Schubert *et al*.^27^. Briefly, spectral data acquired in IDA mode was centroided using qtofpeakpicker^48^. Centroided files were searched with X! Tandem^29^ and Comet^30^ against the annotated yeast proteins database with included reversed decoy peptides. Search results were scored using PeptideProphet and combined with iProphet. Mayu^40^ was used to estimate iProphet probabilities to control for protein identification false discovery rate (FDR <1%). The final spectral library was assembled using SpectraST^49^ by retaining spectra above iProphet FDR controlled cutoff and normalizing chromatography to iRT peptide retention time reference. SpectraST output was then converted to tsv format suitable for Spectronaut retaining 6 most intense transitions of y and b ions using spectrast2tsv from msprotoemicstools^50^. For large-scale data analysis of 327 yeast samples, a minimal consensus library constructed from exhaustion-based IDA acquisitions was compiled in Spectronaut. In the DIA-Umpire approach, we extracted precursor-fragment features by applying signal extraction module from DIA-Umpire workflow using default recommended parameters for TripleTOF5600 instrument on data acquired in SWATH mode by sample exhaustion of the yeast proteome. Generated pseudo MS/MS.mgf files were converted into mzXML and further subjected to database search and processed as described above to generate SWATH assay library. All SWATH data quantification was performed in Spectronaut (v. 8.0.9600, Biognosys) using default settings. Publicly available SWATH libraries used were obtained from the Biognosys library repository or from SWATHAtlas^31^.

For visualization of chromatographic peaks, data of selected peptides was analyzed in Skyline^51^ (v. 3.5.0.9191) with SWATH isolation windows detailed in Suppl. Table 2, and chromatograms of precursors and products exported as text files. For calculation of peak capacities, IDA data was analyzed with QuiC (Biognosys).

Post-processing was conducted in R^52^ by first removing precursors from all samples where the median Qvalue was >0.01. Injection differences were corrected by a robust sum approach where the most and least intense 10% of peptide precursors are removed from the reference pool, and peptides belonging to the same protein were selected based on correlation criteria across all the samples^13^. To account for confounding effects related to acquisition dates in large samples series, we performed batch correction by introducing QC samples in experimental design. External standard QC samples were prepared as a mixture of all injected samples and were measured every 10–12 samples. Each MS acquisition batch had >10 QC samples allowing to correct for the most evident batch effects attributed to an acquisition date (Suppl. Fig. 12). Signal correction was performed using ComBat approach^36^ by specifying covariates that includes QC samples and genotypes of measured mutants using parametric adjustments. Briefly, for every peak group, the signal was modeled as function of the peptide signal and additive and multiplicative batch effects that were estimated by an empirical Bayes framework^36^. The estimated batch effects were then subtracted from the observed signal to obtain a “clean” peptide signal. Finally, the signals of all peptide groups were geometrically averaged. ComBat routine was used as explained in the R sva package documentation^37^. Plotting was performed in ggplot2 package^53^.

### Data availability statement

Supplementary Table 1 gives an overview over the generated datasets; they are further used as basis of a parallel manuscript submission (Zelezniak *et al.*, submitted, *Cell Systems*, December 2017) and enabled the prediction of metabolite concentrations by artificial intelligence. The datasets are being deposited in Pride (https://www.ebi.ac.uk/pride/archive/) and will be made available online upon publication of the manuscript.

## Electronic supplementary material

Supplementary Information

## References

[1] Walzthoeni T, Leitner A, Stengel F, Aebersold R (2013). Mass spectrometry supported determination of protein complex structure. Curr. Opin. Struct. Biol..

[2] Angel TE (2012). Mass spectrometry-based proteomics: existing capabilities and future directions. Chem. Soc. Rev..

[3] Cardoza JD, Parikh JR, Ficarro SB, Marto JA (2012). Mass spectrometry-based proteomics: qualitative identification to activity-based protein profiling. Wiley Interdiscip. Rev. Syst. Biol. Med..

[4] Godoy L, de, Olsen JV, Cox J (2008). Comprehensive mass-spectrometry-based proteome quantification of haploid versus diploid yeast. Nature.

[5] Wilhelm M (2014). Mass-spectrometry-based draft of the human proteome. Nature.

[6] Muntel J (2015). Advancing Urinary Protein Biomarker Discovery by Data-Independent Acquisition on a Quadrupole-Orbitrap Mass Spectrometer. J. Proteome Res..

[7] Gama MR, Collins CH, Bottoli CBG (2013). Nano-liquid chromatography in pharmaceutical and biomedical research. J. Chromatogr. Sci..

[8] Aebersold R, Mann M (2016). Mass-spectrometric exploration of proteome structure and function. Nature.

[9] Röst HL (2016). TRIC: an automated alignment strategy for reproducible protein quantification in targeted proteomics. Nat. Methods.

[10] Soste M (2014). A sentinel protein assay for simultaneously quantifying cellular processes. Nat. Methods.

[11] Uhlen M (2005). A human protein atlas for normal and cancer tissues based on antibody proteomics. Mol. Cell. Proteomics.

[12] Chen G, Weng N-P (2012). Analyzing the phenotypic and functional complexity of lymphocytes using CyTOF (cytometry by time-of-flight). Cell. Mol. Immunol..

[13] Alam MT (2016). The metabolic background is a global player in Saccharomyces gene expression epistasis. Nat Microbiol.

[14] Kustatscher G (2014). Proteomics of a fuzzy organelle: interphase chromatin. EMBO J..

[15] Zou Q, Quan Z (2016). Editorial (Thematic Issue: Machine Learning Techniques for Protein Structure, Genomics Function Analysis and Disease Prediction). Curr. Proteomics.

[16] Kustatscher G, Rappsilber J (2016). Compositional Dynamics: Defining the Fuzzy Cell. Trends Cell Biol.

[17] Plumb RS (2006). UPLC/MSE; a new approach for generating molecular fragment information for biomarker structure elucidation. Rapid Commun. Mass Spectrom..

[18] Gillet LC (2012). Targeted data extraction of the MS/MS spectra generated by data independent acquisition: a new concept for consistent and accurate proteome analysis. Mol. Cell. Proteomics.

[19] Andrews GL, Simons BL, Young JB, Hawkridge AM, Muddiman DC (2011). Performance characteristics of a new hybrid quadrupole time-of-flight tandem mass spectrometer (TripleTOF 5600). Anal. Chem..

[20] Escher C (2012). Using iRT, a normalized retention time for more targeted measurement of peptides. Proteomics.

[21] Ghaemmaghami S (2003). Global analysis of protein expression in yeast. Nature.

[22] Kulak NA, Pichler G, Paron I, Nagaraj N, Mann M (2014). Minimal, encapsulated proteomic-sample processing applied to copy-number estimation in eukaryotic cells. Nat. Methods.

[23] Paulo JA (2016). Quantitative mass spectrometry-based multiplexing compares the abundance of 5000 S. cerevisiae proteins across 10 carbon sources. J. Proteomics.

[24] Nilsson T (2010). Mass spectrometry in high-throughput proteomics: ready for the big time. Nat. Methods.

[25] Gillet LC (2012). Targeted data extraction of the MS/MS spectra generated by data-independent acquisition: a new concept for consistent and accurate proteome analysis. Mol. Cell. Proteomics.

[26] Bruderer R (2015). Extending the limits of quantitative proteome profiling with data-independent acquisition and application to acetaminophen-treated three-dimensional liver microtissues. Mol. Cell. Proteomics.

[27] Schubert OT (2015). Building high-quality assay libraries for targeted analysis of SWATH MS data. Nat. Protoc..

[28] Tsou, C.-C. *et al*. DIA-Umpire: comprehensive computational framework for data-independent acquisition proteomics. *Nat. Methods* **12**, 258–64, 7 p following 264 (2015). 10.1038/nmeth.3255 PMC4399776 25599550

[29] Craig R, Beavis RC (2004). TANDEM: matching proteins with tandem mass spectra. Bioinformatics.

[30] Eng JK, Jahan TA, Hoopmann MR (2013). Comet: an open-source MS/MS sequence database search tool. Proteomics.

[31] Rosenberger G (2014). A repository of assays to quantify 10,000 human proteins by SWATH-MS. Sci Data.

[32] Mülleder M (2012). A prototrophic deletion mutant collection for yeast metabolomics and systems biology. Nat. Biotechnol..

[33] Vowinckel J (2013). The beauty of being (label)-free: sample preparation methods for SWATH-MS and next-generation targeted proteomics. F1000Res..

[34] Gregori J (2012). Batch effects correction improves the sensitivity of significance tests in spectral counting-based comparative discovery proteomics. J. Proteomics.

[35] Scherer, A. Batch Effects and Noise in Microarray Experiments: *Sources and Solutions*. (John Wiley & Sons, 2009).

[36] Johnson WE, Li C, Rabinovic A (2007). Adjusting batch effects in microarray expression data using empirical Bayes methods. Biostatistics.

[37] Leek JT, Johnson WE, Parker HS, Jaffe AE, Storey JD (2012). The sva package for removing batch effects and other unwanted variation in high-throughput experiments. Bioinformatics.

[38] The M, Tasnim A, Käll L (2016). How to talk about protein-level false discovery rates in shotgun proteomics. Proteomics.

[39] Savitski MM, Wilhelm M, Hahne H, Kuster B, Bantscheff M (2015). A Scalable Approach for Protein False Discovery Rate Estimation in Large Proteomic Data Sets. Mol. Cell. Proteomics.

[40] Reiter L (2009). Protein identification false discovery rates for very large proteomics data sets generated by tandem mass spectrometry. Mol. Cell. Proteomics.

[41] Basak T, Bhat A, Malakar D, Pillai M, Sengupta S (2015). In-depth comparative proteomic analysis of yeast proteome using iTRAQ and SWATH based MS. Mol. Biosyst..

[42] Selevsek N (2015). Reproducible and consistent quantification of the Saccharomyces cerevisiae proteome by SWATH-mass spectrometry. Mol. Cell. Proteomics.

[43] Burniston JG, Connolly J, Kainulainen H, Britton SL, Koch LG (2014). Label-free profiling of skeletal muscle using high-definition mass spectrometry. Proteomics.

[44] Teo G (2015). mapDIA: Preprocessing and statistical analysis of quantitative proteomics data from data independent acquisition mass spectrometry. J. Proteomics.

[45] Nygaard V, Rødland EA, Hovig E (2016). Methods that remove batch effects while retaining group differences may lead to exaggerated confidence in downstream analyses. Biostatistics.

[46] Haas R (2017). Designing and interpreting ‘multi-omic’ experiments that may change our understanding of biology. Current Opinion in Systems Biology.

[47] Canelas AB (2010). Integrated multilaboratory systems biology reveals differences in protein metabolism between two reference yeast strains. Nat. Commun..

[48] Chambers MC (2012). A cross-platform toolkit for mass spectrometry and proteomics. Nat. Biotechnol..

[49] Deutsch EW (2010). A guided tour of the Trans-Proteomic Pipeline. Proteomics.

[50] Aebersold, R. *et al*. msproteomicstools. Available at: https://github.com/msproteomicstools. (Accessed: 19th January 2016)

[51] MacLean, B. *et al*. Skyline: an open source document editor for creating and analyzing targeted proteomics experiments. *Bioinformatics* **26**, 966–968 (2010). 10.1093/bioinformatics/btq054 PMC2844992 20147306

[52] R Core Team. R: A Language and Environment for Statistical Computing. (2015).

[53] Wickham, H. ggplot2: Elegant Graphics for Data Analysis. (2009).

