# Supplementary material for: Cost-effective generation of precise label-free quantitative proteomes in high-throughput by microLC and data-independent acquisition
Source: Sci Rep. 2018 Mar 12;8:4346. doi: 10.1038/s41598-018-22610-4 (PMC5847575; doi:10.1038/s41598-018-22610-4)
Supplement: Supplementary file 1 — Supplementary Information [file 41598_2018_22610_MOESM1_ESM.pdf]

Cost-effective generation of precise label-free quantitative proteomes in high-throughput by microLC and data-independent acquisition

Jakob Vowinckel[1,3*], Aleksej Zelezniak[1,4*], Roland Bruderer[3], Michael Mülleder[1,2] Lukas Reiter[3], and Markus Ralser[1,2+]

1 Department of Biochemistry and Cambridge Systems Biology Centre, University of Cambridge, 80 Tennis Court Rd, Cambridge CB2 1GA, UK

2 The Molecular Biology of Metabolism Laboratory, The Francis Crick Institute, 1 Midland Rd, London NW1 1AT, UK

3 Biognosys AG, Wagistrasse 21, CH-8952 Schlieren, Switzerland

4 Department of Biology and Biological Engineering, Chalmers University of Technology, Kemigården 10, SE-412 96, Göteborg, Sweden

+ To whom correspondence should be addressed. markus.ralser@crick.ac.uk, Tel +44 1223 761346

- These authors contributed equally to this work.

Suppl. Fig. 1. Accumulation time in microLC-SWATH on a TripleTOF5600

Precursor and product intensities, exemplified by the yeast *SSB1* peptide SSNITISNAVGR, were determined at 3 µl/min flow rate in SWATH acquisition. Accumulation times 40 - 75 ms do not have a significant impact on signal intensity of precursors (solid line) or products (shaded lines). Number of quantifiable proteins in microLC-MS is not reduced by accumulation time down to 40 ms (inset).

Suppl. Fig. 2. Distribution of peptide precursors in the yeast proteome.

Precursors identified in the sample fractionation SWATH library approach were plotted according to their mass-to-charge ratio and coloured according to the number of amino acids. 26868 out of 32754 peptides (82 %) are between 400 and 850 m/z, while 31408 (96 %) are between 400 and 1250 m/z.

Suppl. Fig. 3. Yeast protein quantification from microLC-SWATH using 29 x 16 m/z isolation windows.

A yeast tryptic digest was analysed using microLC (25 cm x 0.3 mm Triart-C18, 3 µl/min, 60 min gradient) and coupled to a TripleTOF5600 MS operating in SWATH mode by repeated injection of 10 µg yeast proteome

digest (9x). Isolation windows were chosen as 29x16 m/z, with 40 ms accumulation time and a mass range of 400-850 m/z.
Data was processed with Spectronaut v8.0 using SWATH libraries generated a) by sample fractionation (*frac*) or b) sample exhaustion (*exh*), c) using a publicly accessible spectral library (*Biognosys library*, Spectronaut repository), or with a library generated by DIA-Umpire. Library a) allowed quantification of 1422 proteins, while 1157 proteins could be quantified using library b). The public library (c) did quantify 1118, and DIA-Umpire, that uses correlation patterns to create a library directly out of the SWATH data, 890 proteins. Spectral libraries (except the one obtained from Spectronaut repository) were generated according to Schubert et al.[1]

Suppl. Fig. 4. Yeast peptide quantification from microLC-SWATH using 34 x 25 m/z isolation windows.

A yeast tryptic digest was analysed using microLC (25 cm x 0.3 mm Triart-C18, 3 µl/min, 60 min gradient) and coupled to a TripleTOF5600 MS operating in SWATH mode by repeated injection of 10 µg yeast proteome digest (9x). Isolation windows were chosen as 34x25 m/z, with 100 ms accumulation time and a mass range of 400-1250 m/z.
Data was processed with Spectronaut v8.0 using SWATH libraries generated a) by sample fractionation (*frac*) or b) sample exhaustion (*exh*), c) using a publicly accessible spectral library (*Biognosys library*, Spectronaut repository), or with a library generated by DIA-Umpire. Library a) allowed quantification of 8824 peptides, while 6866 peptides could be quantified using library b). The public library (c) did quantify 8283, and DIA-Umpire, that uses correlation patterns to create a library directly out of the SWATH data, 6132 peptides. Spectral libraries (except the one obtained from Spectronaut repository) were generated according to Schubert et al.[1]

Suppl. Fig. 5. Yeast peptide quantification from microLC-SWATH using 29 x 16 m/z isolation windows.

A yeast tryptic digest was analysed using microLC (25 cm x 0.3 mm Triart-C18, 3 µl/min, 60 min gradient) and coupled to a TripleTOF5600 MS operating in SWATH mode by repeated injection of 10 µg yeast proteome digest (9x). Isolation windows were chosen as 29x16 m/z, with 40 ms accumulation time and a mass range of 400-850 m/z.
Data was processed with Spectronaut v8.0 using SWATH libraries generated a) by sample fractionation (*frac*) or b) sample exhaustion (*exh*), c) using a publicly accessible spectral library (*Biognosys library*, Spectronaut repository), or with a library generated by DIA-Umpire. Library a) allowed quantification of 6598 peptides, while 5673 peptides could be quantified using library b). The public library (c) did quantify 6518, and DIA-Umpire, that uses correlation patterns to create a library directly out of the SWATH data, 4841 peptides. Spectral libraries (except the one obtained from Spectronaut repository) were generated according to Schubert et al.[1]

Suppl. Fig. 6. Technical variability of yeast peptide quantification is low in microLC-SWATH-MS irrespective of data extraction

Fold change variability of 765 peptide precursors present in all data sets was compared throughout nine replicates. Median coefficients of variation are between 7.3 % and 8.4 % for libraries generated using respectively fractionation and exhaustion approach, 8.8 % for an unrelated yeast library, and 7 % for a library generated by DIA-Umpire. Spectral libraries (except the one obtained from Spectronaut repository) were generated according to Schubert et al.[1]

Suppl. Fig. 7. Human peptide quantification from microLC-SWATH data.

A tryptic digest of a whole-cell protein extract from human K562 cells was analysed using microLC (25 cm x 0.3 mm Triart-C18, 3 µl/min, 60 min gradient) and coupled to a TripleTOF5600 MS operating in SWATH mode by repeated injection of 3 µg digest (6x). Data was processed with Spectronaut v8.0 using a SWATH library obtained from the SWATHAtlas repository[2] (*10k library*), or using SWATH libraries generated by repeated analysis of HEK293 or HeLa cell extracts (Spectronaut repository). Data analysis using a rich library allows quantification of 20508 peptides, while 9256 peptides can be quantified using a HEK293 and 12272 using a HeLa library, respectively.

Suppl. Fig. 8. Technical variability of human peptide quantification is low in microLC-SWATH-MS irrespective of data extraction

Fold change variability of 6722 peptide precursors present in all data sets compared throughout the nine replicates. Median coefficients of variation of signal intensities are between 5.8 and 6.5 %, for all libraries.

Suppl. Fig. 9. Direct correlation of peptide intensities between two human samples reveals no negative bias towards low intensity peptides

Correlation of peptide intensities of two human samples is linear (R^2 = 0.98), and correlation is equally good for high and low intensity peptides over five orders of magnitude.

Suppl. Fig. 10. Coefficients of variation show little bias towards low intensity peptides

Coefficients of variation of peptide intensities of six human samples is low (mean 3-10 %) for peptides with intensities >1000 cps, and only marginally elevated for lower peptides (mean 8-12 %). Inset: Coefficients of variation of peptide intensities of six human samples.

Suppl. Fig. 11. Library size is associated to quantification precision.

Protein identifications in a spectral library generated by sample fractionation (green; 3896 proteins) or using DIA-UMPIRE approach (violet; 853 proteins) were plotted against absolute protein abundances (log scale) reported by two independent datasets (Kulak et al. 2014[3]; Ghaemmaghami et al. 2003[4]). DIA-Umpire library indeed captures highly abundant proteins.

Suppl. Fig. 12. Total precursor intensities before and after batch correction

Combined intensities of all precursors varies with experimental batches, and is equalled out by batch correction.

Suppl. Fig. 13. Protein fold change standard deviation is reduced by batch correction

Protein fold change was calculated from three most abundant peptides of the representative ZRP1_YEAST protein quantified in two yeast strains in 9

replicates (3 batches). After batch correction, variability is considerably reduced.

Suppl. Fig. 1

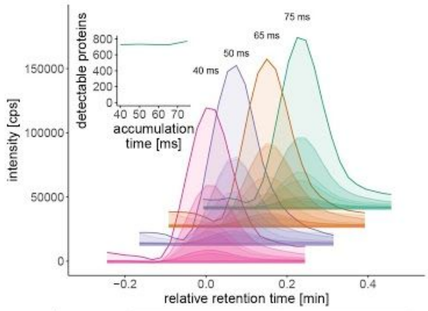

Suppl. Fig. 2

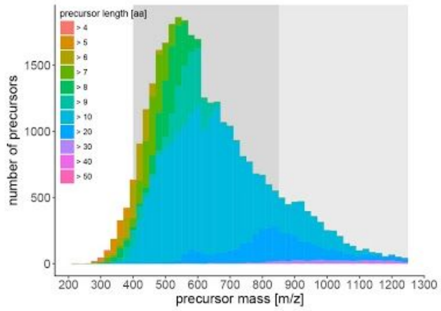

Suppl. Fig. 3

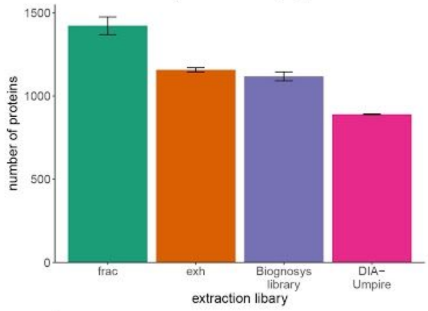

Suppl. Fig. 4

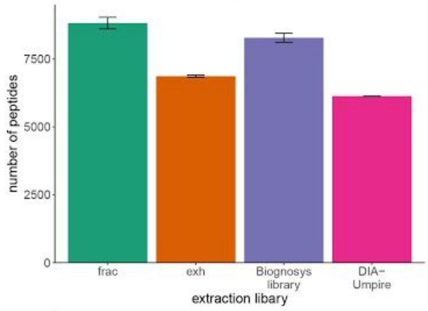

Suppl. Fig. 5

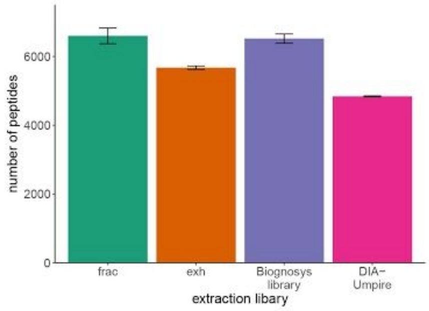

Suppl. Fig. 6

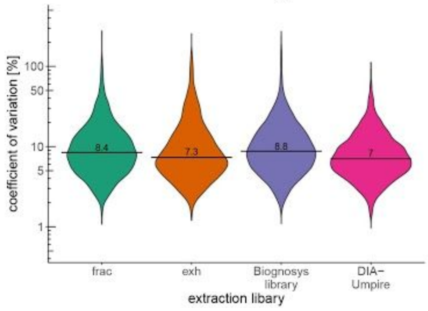

Suppl. Fig. 7

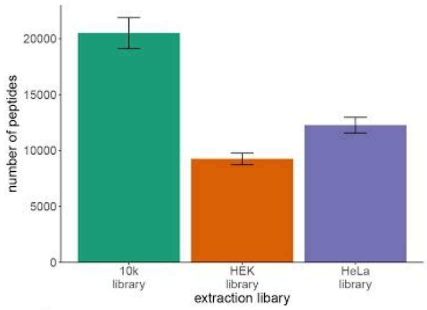

Suppl. Fig. 8

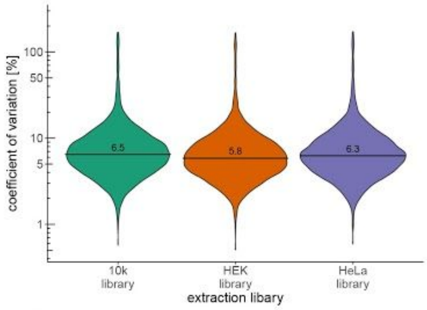

Suppl. Fig. 9

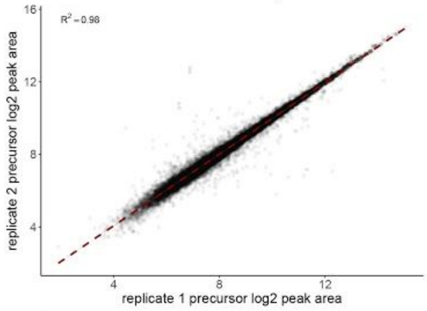

Suppl. Fig. 10

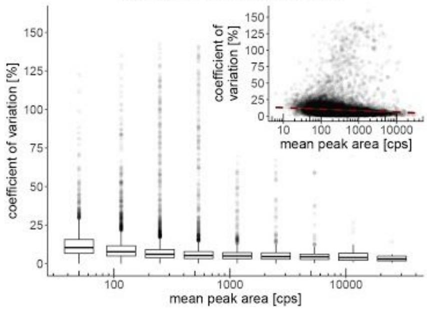

Suppl. Fig. 11

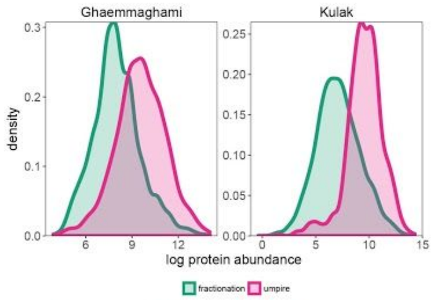

Suppl. Fig. 12

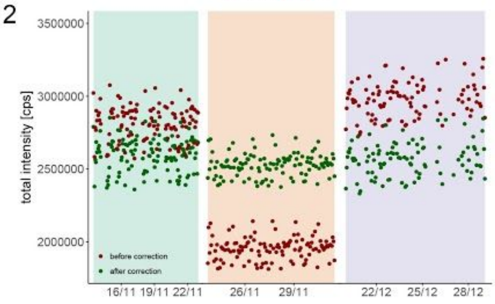

Suppl. Fig. 13

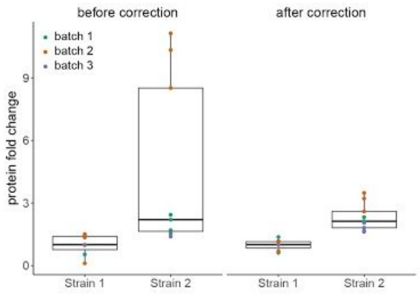

Supplementary Tables

Suppl. table 1: Data sets included in this study

Figure panel	Sample	Experiment	Acquisition mode
A-D	Yeast lysate	Multiple chromatography regimes	DDA
E	Yeast lysate	60 min water to acetonitrile gradient at a flow-rate of 3 µL/min, varying amounts of loaded protein	DDA
F	Yeast lysate	5 µg yeast protein injected and separated using water-to-acetonitrile chromatographic gradients of 10-90 min at a flow-rate of 3 µL/min	DDA
G	Yeast lysate	The sample first separated by high pH reverse phase chromatography on an analytical HPLC and then each fraction is analyzed with 3 µl/min flow rate. In the sample exhaustion approach, the same digest was instead injected repeatedly until protein identification was saturated.	DDA
H	Yeast lysate	SWATH using 34 x 25 m/z or 29 x 16 m/z windows at 3 µl/min flowrate. Later used for acquisition of 397 yeast samples in (Zelezniak et al, parallel submission)	DIA
I	Yeast lysate	For fractionation and exhaustion library same samples as G. DIA-UMPIRE library created using data from 9 injections recorded with 34 x 25 m/z width SWATH windows	DDA/DIA
J, L	human K562 cells lysate	6 injections, 3 µl/min, 60 min gradient, 3 µg tryptic digest with 34 x 25 m/z width SWATH windows, libraries from SWATHAtlas repository	DIA
K	Yeast lysate	9 injections, 3 µl/min, 60 min gradient recorded with 10 µg tryptic digest 34 x 25 m/z width SWATH windows	DIA

M-R	Yeast lysate	296 proteomes, grown in 9 replicates (3 biological x 3 technical) recorded with 34 x 25 m/z width SWATH windows, 3 µl/min, 60 min gradient	DIA

Suppl. table 2: Chromatographic gradients

Gradient	min	%A
**10 min**	1	97
	8.3	60
	10	60
	15	97
	27	97
**20 min**	1	97
	16.7	60
	20	60
	25	97
	37	97
**30 min**	1	97
	25	60
	30	60
	35	97
	47	97
**45 min**	1	97
	37.5	60
	45	60
	50	97
	62	97
**60 min**	1	97
	50	60
	60	60
	65	97
	77	97
**90 min**	1	97
	75	60
	90	60
	95	97
	107	97

Suppl. table 3: SWATH isolation windows

34x25 m/z regime

Start	End	Overlap	acc. time
400	425	1	100
425	450	1	100
450	475	1	100
475	500	1	100
500	525	1	100
525	550	1	100
550	575	1	100
575	600	1	100
600	625	1	100
625	650	1	100
650	675	1	100
675	700	1	100
700	725	1	100
725	750	1	100
750	775	1	100
775	800	1	100
800	825	1	100
825	850	1	100
850	875	1	100
875	900	1	100
900	925	1	100
925	950	1	100
950	975	1	100
975	1000	1	100
1000	1025	1	100
1025	1050	1	100
1050	1075	1	100
1075	1100	1	100
1100	1125	1	100
1125	1150	1	100
1150	1175	1	100
1175	1200	1	100
1200	1225	1	100
1225	1250	1	100

29x16 m/z regime

Start	End	Overlap	acc time
400	416	1	40
416	432	1	40
432	448	1	40
448	464	1	40
464	480	1	40
480	496	1	40
496	512	1	40
512	528	1	40
528	544	1	40
544	560	1	40
560	576	1	40
576	592	1	40
592	608	1	40
608	624	1	40
624	640	1	40
640	656	1	40
656	672	1	40
672	688	1	40
688	704	1	40
704	720	1	40
720	736	1	40
736	752	1	40
752	768	1	40
768	784	1	40
784	800	1	40
800	816	1	40
816	832	1	40
832	848	1	40
848	850	1	40

(1) Schubert, O. T.; Gillet, L. C.; Collins, B. C.; Navarro, P.; Rosenberger, G.; Wolski, W. E.; Lam, H.; Amodei, D.; Mallick, P.; MacLean, B.; Aebersold, R. *Nat. Protoc.* **2015**, *10* (3), 426–441.

(2) Rosenberger, G.; Koh, C. C.; Guo, T.; Röst, H. L.; Kouvonen, P.; Collins, B. C.; Heusel, M.; Liu, Y.; Caron, E.; Vichalkovski, A.; Faini, M.; Schubert, O. T.; Faridi, P.; Ebhardt, H. A.; Matondo, M.; Lam, H.; Bader, S. L.; Campbell, D. S.; Deutsch, E. W.; Moritz, R. L.; Tate, S.; Aebersold, R. *Sci Data* **2014**, *1*, 140031.

(3) Kulak, N. A.; Pichler, G.; Paron, I.; Nagaraj, N.; Mann, M. *Nat. Methods* **2014**, *11* (3), 319–324.

(4) Ghaemmaghami, S.; Huh, W.-K.; Bower, K.; Howson, R. W.; Belle, A.; Dephoure, N.; O'Shea, E. K.; Weissman, J. S. *Nature* **2003**, *425* (6959), 737–741.
